# Development of an interpretable machine learning model for predicting in-hospital mortality in ICU patients with sepsis: a retrospective cohort study

**DOI:** 10.3389/fmed.2026.1889209

**Published:** 2026-09-15

**Authors:** Tianyu Zhao, Kexin Wen, Xumin Han, Ruimin Tan, Yongtao Wang, Quansheng Du

**Affiliations:** 1 Department of Emergency Medicine and Critical Care Medicine (Ward 2), Hebei General Hospital, Shijiazhuang, Hebei, China; 2 Hebei North University, Zhangjiakou, China; 3 Hebei Medical University, Shijiazhuang, China; 4 Shenzhen Second People's Hospital, Shenzhen, China; 5 Department of Anesthesiology, Shengli Clinical Medical College of Fujian Medical University, Fujian Provincial Hospital, Fuzhou, China; 6 Handan First Hospital, HanDan, China

**Keywords:** in-hospital mortality, machine learning, random forest, sepsis, shap

## Abstract

**Background:**

Sepsis ranks among the primary causes of mortality in intensive care units (ICUs), often resulting in multiple organ dysfunction due to dysregulated systemic inflammation. Timely recognition of patients at high risk is essential, yet existing clinical scoring systems show limited predictive performance. We aimed to design and evaluate an interpretable machine learning model to predict the risk of in-hospital mortality in individuals with sepsis.

**Methods:**

This retrospective study screened 503 sepsis patients who were admitted to the ICU at Hebei Provincial People's Hospital from January 2022 through January 2025. After excluding patients with ICU stay less than 24 h, incomplete clinical data, or pregnancy, 431 patients were included. The dataset was randomly partitioned into a training set (*n* = 301) and a validation set (*n* = 130). Eight machine learning algorithms—including logistic regression, naive Bayes, support vector machine, single-layer neural network, random forest (RF), LightGBM, K-nearest neighbors, and decision tree—were trained and evaluated. Model performance was assessed by ROC curves (AUC), accuracy, F1 score, sensitivity, specificity, Brier score, expected calibration error, and decision curve analysis. The best-performing model was interpreted using SHAP values.

**Results:**

Among 431 patients, 198 (45.9%) died during hospitalization. Feature selection using the Boruta algorithm identified 12 key predictors. The RF model achieved the most balanced overall performance, with an area under the ROC curve of 0.804 (95% CI, 0.730–0.879) in the validation set, accuracy of 0.785, F1 score of 0.778, sensitivity of 0.817, and specificity of 0.757. SHAP analysis identified lactate dehydrogenase and urea as the most influential predictors, followed by age, APACHE II score, and procalcitonin. An interactive web application was developed as a research prototype to demonstrate the feasibility of model deployment.

**Conclusions:**

We developed and internally validated an interpretable RF-based machine learning model for predicting in-hospital mortality in patients with sepsis. The model showed acceptable discrimination, reasonable calibration, and potential clinical net benefit in internal validation. SHAP-based interpretation provided insight into key predictive factors, while the web-based calculator demonstrated the feasibility of model deployment. However, external and prospective validation is required before its clinical utility and generalizability can be established.

## Introduction

Sepsis occurs when an abnormal host response to infection causes life-threatening organ dysfunction and remains a major challenge in intensive care (1). Globally, it affects roughly 50 million individuals each year and is responsible for about 11 million deaths, accounting for nearly 20% of all mortalities, with the highest incidence and severity observed in intensive care units (ICUs) (2, 3). Despite advances in supportive care, early antimicrobial therapy, and organ support, sepsis-related mortality remains high, particularly among older patients and those with substantial comorbidity or severe organ dysfunction at admission (3, 4).

Sepsis pathophysiology reflects intricate interactions between pathogen virulence and the host immune system, leading to endothelial injury, microcirculatory collapse, and abnormal coagulation, which together increase the risk of multi-organ failure (5). This complexity renders early risk stratification difficult, as clinical signs may lag underlying systemic derangements. Traditional severity scores such as the SOFA, APACHE II, and SAPS II were developed to quantify illness severity and predict mortality risk (6–8). While widely adopted, these scoring systems have several limitations: they were not designed as individualized prognostic tools, often demonstrate only moderate discrimination in contemporary sepsis cohorts, rely on static threshold cutoffs rather than dynamic risk patterns, and their performance can vary substantially across different healthcare settings (9).

Given these limitations, there has been increasing interest in applying machine learning (ML) to prognostic modeling in sepsis, capitalizing on its ability to integrate large numbers of heterogeneous clinical variables, capture complex nonlinear interactions, and iteratively improve predictive performance (10, 11). However, many ML models remain insufficiently interpretable, often described as “black boxes,” which impedes clinical acceptance and integration into care pathways.

To address this gap, interpretable ML techniques such as SHAP and LIME have been introduced, enabling quantification of individual feature contributions and offering clinicians insights into how specific variables influence model predictions (12, 13). Recent studies have applied these approaches to sepsis prognosis. For example, Hu et al. developed SHAP-based interpretable models using MIMIC-IV data, while Park et al. evaluated explainable machine learning in a multicenter sepsis cohort (14, 15). Despite these advances, important limitations remain, including insufficient external validation, heterogeneity in predictor selection and modeling strategies, and uncertain generalizability across different healthcare settings. These concerns may be particularly relevant to Chinese tertiary ICUs, where patient characteristics and clinical practice patterns may differ from those of Western cohorts (16, 17). Therefore, in the present study, we systematically compared eight machine learning algorithms using routinely available variables collected within the first 24 h of ICU admission and further benchmarked the final model against SOFA and APACHE II scores.

In this context, there is a pressing need for locally validated, interpretable prognostic models that combine the predictive power of ML with transparency and actionable insights for bedside use. We conducted a retrospective cohort study of ICU patients with sepsis at Hebei Provincial People's Hospital, applying a suite of ML algorithms with robust feature selection and SHAP interpretability. Our goal was to develop and validate a model capable of accurately predicting in-hospital mortality, elucidate key driving risk factors, and provide a framework for clinical implementation that bridges predictive performance with interpretability.

## Materials and methods

### Study design and population

This retrospective cohort study was conducted in the Department of Critical Care Medicine of Hebei Provincial People's Hospital. Adult patients with sepsis admitted to the ICU between January 2022 and January 2025 were screened. Sepsis was diagnosed according to the Sepsis-3 criteria, defined as suspected or documented infection accompanied by an acute increase in SOFA score of at least 2 points (1). Patients were eligible if they met the Sepsis-3 diagnostic criteria. Patients were excluded if they were ≤18 years old, had an ICU stay <24 h, had incomplete required clinical data or unclear clinical outcomes, or were pregnant or lactating.

This study was approved by the Ethics Committee of Hebei Provincial People's Hospital (approval No. 2026-LW-002), and due to its retrospective design, the need for written informed consent was waived. Initially, 503 patients were screened, and after applying the exclusion criteria, 431 patients were included in the study, consisting of 233 survivors and 198 non-survivors. The cohort was randomly split into a training set (*n* = 301, 70%) and a validation set (*n* = 130, 30%). The patient selection and model development workflow is shown in Figure 1.

**Figure 1:**
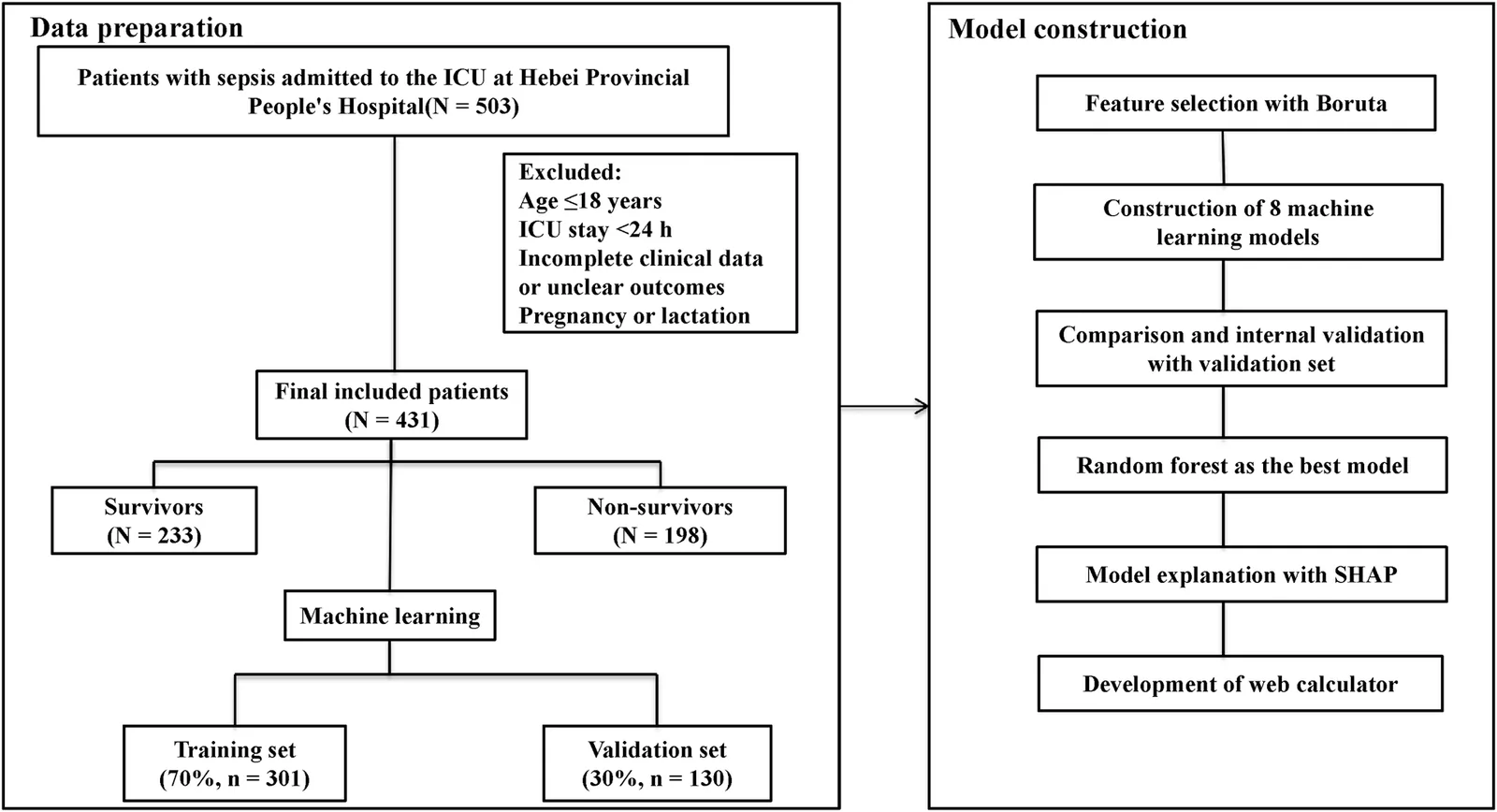
Flow chart of patient selection and model development. ICU, intensive care unit; SHAP, SHapley Additive exPlanations.

### Outcome

The primary endpoint of the study was in-hospital mortality, defined as death from any cause occurring during the index hospitalization. Based on this outcome, patients were categorized into survivor and non-survivor groups.

### Data collection

Data were extracted from the electronic medical record system. Candidate predictors included demographics, vital signs, comorbidities, laboratory measurements, treatment-related variables, and severity scores. ICU admission was defined as the index time, and all applicable candidate predictors were restricted to information available within the first 24 h after ICU admission. Demographic and vital sign variables included sex, age, systolic and diastolic blood pressure, and mean arterial pressure. Comorbidities included hypertension, diabetes mellitus, coronary heart disease, cerebrovascular disease, respiratory disease, and malignancy. Treatment-related variables comprised vasoactive drug use, continuous renal replacement therapy (CRRT), and mechanical ventilation administered within the first 24 h after ICU admission. Severity of illness was represented by SOFA and APACHE II scores. Laboratory variables included procalcitonin, white blood cell count, neutrophil count, lymphocyte count, hemoglobin, platelet count, total protein, albumin, bilirubin indices, liver enzymes, renal function markers, lactate dehydrogenase, creatine kinase, creatine kinase-MB isoenzyme, lactate, oxygenation index, and coagulation parameters including PT, INR, APTT, and TT.

### Missing data

The proportion of missing data was assessed for all candidate predictors before analysis. All included variables had less than 20% missingness and were therefore retained for subsequent analysis (Supplementary Table S1). Missing data were handled using multiple imputation by chained equations with five imputations (*m* = 5). Imputation and preprocessing were performed within the training framework to minimize information leakage.

### Feature selection and model development

A total of 39 candidate predictors were considered for feature selection. Boruta, a random forest–based selection algorithm, was applied only to the training set to identify predictors of in-hospital mortality. Variables confirmed by Boruta were retained for model construction. Eight supervised machine learning models were developed: logistic regression (LR), naive Bayes (NB), support vector machine (SVM), single-layer hidden-layer neural network (SHLNN), random forest (RF), Light Gradient Boosting Machine(LightGBM), K-nearest neighbors (KNN), and decision tree (DT). Feature selection, model training, and hyperparameter tuning were conducted using the training set, while the validation set was reserved solely for independent evaluation of model performance. During training, repeated 5-fold cross-validation with two repeats was performed, and the area under the receiver operating characteristic curve (AUC) was used as the primary metric for model tuning and comparison. Hyperparameters were optimized within the training set during repeated 5-fold cross-validation, and the final settings for each algorithm are provided in Supplementary Table S2. The outcome distribution was relatively balanced; therefore, no class weighting, oversampling, undersampling, or synthetic sampling methods were applied during model training.

### Model evaluation

Model performance was evaluated in the training and validation sets. In the training set, internal validation was based on out-of-fold predicted probabilities generated during cross-validation. Discrimination was assessed using ROC curves and AUC values, with 95% confidence intervals (CIs) for the AUCs estimated using the DeLong method. To further assess the discriminative performance of the final model relative to established clinical severity scores, ROC curves for the SOFA and APACHE II scores were generated separately in the validation set. The AUCs of the final RF model, SOFA score, and APACHE II score were compared using paired DeLong tests. Classification performance was evaluated using sensitivity, specificity, PPV, NPV, accuracy, and F1 score. Calibration was assessed using calibration curves, Brier score, and expected calibration error (ECE), with ECE calculated using 10 fixed probability bins. Decision curve analysis was employed to evaluate clinical applicability. The final model was selected based on its overall balance across discrimination, classification performance, calibration, and clinical net benefit rather than on any single performance metric.

### Model interpretation and web application

SHAP was applied to the best-performing model to evaluate feature importance, illustrate the impact and direction of each predictor, and provide individualized explanations for selected patients. To facilitate clinical use, the final model was deployed as an interactive web-based application using Python and Streamlit. Users can input selected clinical predictors through a browser-based interface and obtain the estimated probability of in-hospital mortality.

### Statistical analysis

Continuous data are reported as mean ± standard deviation or median with interquartile range, as appropriate, and categorical data as counts with percentages. Between-group differences were evaluated using the independent-samples *t* test, Wilcoxon rank-sum test, chi-square test, or Fisher's exact test. Two-sided *P* values < 0.05 were considered statistically significant. Statistical analyses were performed using R software version 4.5.0. Machine learning model development, validation, SHAP analysis, and web application deployment were performed using Python version 3.12.7.

## Results

### Patient characteristics and dataset partitioning

Baseline characteristics stratified by survival status are shown in Table 1. Non-survivors were older, had higher SOFA and APACHE II scores, and more commonly received vasoactive agents, CRRT, and mechanical ventilation. Regarding comorbidities, hypertension, coronary heart disease, and respiratory disease were more frequent in the non-survivor group. For laboratory variables, non-survivors had lower lymphocyte counts and higher aspartate aminotransferase, urea, creatinine, lactate dehydrogenase, and thrombin time levels. Procalcitonin also differed significantly between groups, with a higher median value observed in survivors. No significant differences were found in sex, MAP, diabetes mellitus, cerebrovascular disease, malignant tumor, WBC, neutrophil count, hemoglobin, platelet count, total protein, albumin, direct bilirubin, ALT, CK-MB isoenzyme, lactate, oxygenation index, PT, INR, or APTT.

**Table 1:** Baseline characteristics of survivors and non-survivors among patients with sepsis.

Variables	Total (*n* = 431)	Survivors (*n* = 233)	Non-survivors (*n* = 198)	*P*
Sex, *n* (%)				0.933
Male	298 (69.10)	162 (69.50)	136 (68.70)	
Female	133 (30.90)	71 (30.50)	62 (31.30)	
Age, years	73.00 (62.50, 82.00)	69.00 (58.00, 80.00)	76.00 (69.00, 85.75)	<0.001
SBP, mmHg	122.00 (105.00, 141.50)	122.00 (105.00, 138.00)	123.50 (106.00, 144.50)	0.468
DBP, mmHg	66.00 (56.00, 78.00)	67.00 (58.00, 81.00)	64.50 (55.00, 73.75)	0.016
MAP, mmHg	83.00 (73.00, 95.50)	85.00 (73.00, 99.00)	83.00 (73.00, 93.00)	0.156
SOFA score	7.00 (5.00, 10.00)	7.00 (5.00, 9.00)	8.00 (6.00, 12.00)	<0.001
APACHE II score	21.00 (17.00, 27.00)	19.00 (14.00, 24.00)	24.00 (19.00, 29.00)	<0.001
Use of vasoactive drugs, *n* (%)				<0.001
Yes	380 (88.20)	189 (81.10)	191 (96.50)	
No	51 (11.80)	44 (18.90)	7 (3.50)	
Use of CRRT, *n* (%)				<0.001
Yes	124 (28.80)	41 (17.60)	83 (41.90)	
No	307 (71.20)	192 (82.40)	115 (58.10)	
Mechanical ventilation, *n* (%)				0.001
Yes	399 (92.60)	206 (88.40)	193 (97.50)	
No	32 (7.40)	27 (11.60)	5 (2.50)	
Hypertension, *n* (%)				0.029
Yes	216 (50.10)	105 (45.10)	111 (56.10)	
No	215 (49.90)	128 (54.90)	87 (43.90)	
Diabetes mellitus, *n* (%)				0.153
Yes	130 (30.20)	63 (27.00)	67 (33.80)	
No	301 (69.80)	170 (73.00)	131 (66.20)	
Coronary heart disease, *n* (%)				0.001
Yes	128 (29.70)	53 (22.70)	75 (37.90)	
No	303 (70.30)	180 (77.30)	123 (62.10)	
Cerebrovascular disease, *n* (%)				0.285
Yes	170 (39.40)	86 (36.90)	84 (42.40)	
No	261 (60.60)	147 (63.10)	114 (57.60)	
Respiratory disease, *n* (%)				0.003
Yes	301 (69.80)	148 (63.50)	153 (77.30)	
No	130 (30.20)	85 (36.50)	45 (22.70)	
Malignant tumor, *n* (%)				1.000
Yes	42 (9.70)	23 (9.90)	19 (9.60)	
No	389 (90.30)	210 (90.10)	179 (90.40)	
Procalcitonin, ng/mL	3.96 (0.78, 23.66)	5.63 (1.24, 27.75)	2.30 (0.58, 15.45)	<0.001
White blood cell count, ×10^9^/L	13.50 (9.61, 19.28)	13.75 (9.61, 19.71)	13.38 (9.69, 18.89)	0.362
Neutrophil count, ×10^9^/L	12.07 (8.28, 17.32)	11.82 (8.25, 18.06)	12.19 (8.31, 17.04)	0.644
Lymphocyte count, ×10^9^/L	0.73 (0.43, 1.17)	0.79 (0.48, 1.33)	0.62 (0.39, 1.01)	0.001
Hemoglobin, g/L	99.00 (83.00, 117.00)	102.00 (84.00, 117.00)	95.50 (80.25, 116.00)	0.140
Platelet count, ×10^9^/L	169.00 (91.50, 228.00)	174.00 (92.00, 238.00)	158.50 (89.25, 218.00)	0.312
Total protein, g/L	49.93 ± 10.37	49.38 ± 10.32	50.59 ± 10.41	0.227
Albumin, g/L	25.95 ± 5.18	26.06 ± 5.23	25.82 ± 5.14	0.633
Total bilirubin, μmol/L	16.10 (10.60, 25.25)	17.00 (11.40, 26.10)	14.70 (10.03, 24.25)	0.055
Direct bilirubin, μmol/L	5.80 (3.10, 10.90)	6.10 (3.20, 11.20)	5.05 (3.10, 10.32)	0.228
ALT, U/L	25.20 (13.15, 54.80)	25.40 (12.80, 59.30)	24.90 (13.35, 53.48)	0.928
AST, U/L	39.40 (23.70, 88.10)	36.70 (21.90, 84.50)	43.55 (25.17, 95.62)	0.047
Urea, mmol/L	11.30 (7.60, 18.50)	9.30 (6.70, 14.40)	13.98 (8.70, 22.05)	<0.001
Creatinine, μmol/L	97.00 (65.75, 161.30)	91.80 (63.30, 141.20)	111.95 (71.80, 188.93)	0.007
LDH, U/L	296.40 (217.35, 467.65)	262.20 (200.10, 388.80)	375.40 (255.02, 570.45)	<0.001
Creatine kinase, U/L	127.50 (46.85, 360.75)	160.00 (53.10, 385.00)	93.95 (40.25, 296.50)	0.026
CK-MB, IU/L	16.70 (11.15, 28.80)	16.70 (10.70, 29.40)	16.65 (11.85, 27.92)	0.365
Lactate, mmol/L	2.80 (1.86, 5.12)	2.90 (1.74, 4.70)	2.73 (1.90, 5.48)	0.161
Oxygenation index, mmHg	172.35 (97.64, 229.58)	174.79 (106.48, 240.54)	159.54 (88.43, 222.75)	0.063
PT, s	13.70 (12.30, 16.50)	13.50 (12.40, 15.60)	14.00 (12.30, 17.10)	0.147
INR	1.19 (1.08, 1.45)	1.18 (1.08, 1.37)	1.23 (1.07, 1.51)	0.167
APTT, s	32.00 (28.20, 37.95)	31.20 (27.90, 36.80)	32.70 (28.50, 39.27)	0.083
TT, s	16.50 (15.50, 18.10)	16.50 (15.20, 17.90)	16.65 (15.80, 18.50)	0.028

SBP, systolic blood pressure; DBP, diastolic blood pressure; MAP, mean arterial pressure; SOFA, Sequential Organ Failure Assessment; APACHE II, Acute Physiology and Chronic Health Evaluation II; CRRT, continuous renal replacement therapy; ALT, alanine aminotransferase; AST, aspartate aminotransferase; LDH, lactate dehydrogenase; CK-MB, creatine kinase-MB isoenzyme; PT, prothrombin time; INR, international normalized ratio; APTT, activated partial thromboplastin time; TT, thrombin time.

The cohort was randomly partitioned into training and validation sets at a 70:30 ratio using stratification by mortality status, resulting in 301 patients in the training set and 130 in the validation set. The distribution of in-hospital mortality was similar between the training and validation sets, with 138 deaths in the training set and 60 deaths in the validation set. As shown in Table 2, most demographic characteristics, vital signs, comorbidities, treatment-related variables, severity scores, and laboratory variables were well balanced between the two sets. Age showed a marginal but non-significant difference between the training and validation sets. Overall, the two sets were sufficiently comparable for subsequent model training and validation.

**Table 2:** Baseline characteristics of the training and validation sets.

Variables	Total (*n* = 431)	Training set (*n* = 301)	Validation set (*n* = 130)	*P*
Sex, *n* (%)				0.133
Male	298 (69.10)	201 (66.80)	97 (74.60)	
Female	133 (30.90)	100 (33.20)	33 (25.40)	
Age, years	73.00 (62.50, 82.00)	74.00 (64.00, 85.00)	69.50 (59.00, 79.00)	0.052
In-hospital mortality, *n* (%)				1.000
Yes	198 (45.90)	138 (45.80)	60 (46.20)	
No	233 (54.10)	163 (54.20)	70 (53.80)	
SBP, mmHg	122.00 (105.00, 141.50)	122.00 (105.00, 144.00)	122.00 (108.00, 136.75)	0.876
DBP, mmHg	66.00 (56.00, 78.00)	66.00 (57.00, 79.00)	65.00 (55.00, 75.75)	0.445
MAP, mmHg	83.00 (73.00, 95.50)	83.00 (73.00, 98.00)	86.00 (73.00, 93.00)	0.598
SOFA score	7.00 (5.00, 10.00)	7.00 (5.00, 10.00)	7.50 (5.00, 10.00)	0.534
APACHE II score	21.77 ± 7.86	22.07 ± 7.91	21.08 ± 7.71	0.227
Use of vasoactive drugs, *n* (%)				0.774
Yes	380 (88.20)	264 (87.70)	116 (89.20)	
No	51 (11.80)	37 (12.30)	14 (10.80)	
Use of CRRT, *n* (%)				0.834
Yes	124 (28.80)	88 (29.20)	36 (27.70)	
No	307 (71.20)	213 (70.80)	94 (72.30)	
Mechanical ventilation, *n* (%)				0.123
Yes	399 (92.60)	283 (94.00)	116 (89.20)	
No	32 (7.40)	18 (6.00)	14 (10.80)	
Hypertension, *n* (%)				1.000
Yes	216 (50.10)	151 (50.20)	65 (50.00)	
No	215 (49.90)	150 (49.80)	65 (50.00)	
Diabetes mellitus, *n* (%)				1.000
Yes	130 (30.20)	91 (30.20)	39 (30.00)	
No	301 (69.80)	210 (69.80)	91 (70.00)	
Coronary heart disease, *n* (%)				0.475
Yes	128 (29.70)	93 (30.90)	35 (26.90)	
No	303 (70.30)	208 (69.10)	95 (73.10)	
Cerebrovascular disease, *n* (%)				1.000
Yes	170 (39.40)	119 (39.50)	51 (39.20)	
No	261 (60.60)	182 (60.50)	79 (60.80)	
Respiratory disease, *n* (%)				0.396
Yes	301 (69.80)	206 (68.40)	95 (73.10)	
No	130 (30.20)	95 (31.60)	35 (26.90)	
Malignant tumor, *n* (%)				1.000
Yes	42 (9.70)	29 (9.60)	13 (10.00)	
No	389 (90.30)	272 (90.40)	117 (90.00)	
Procalcitonin, ng/mL	3.96 (0.78, 23.66)	3.59 (0.77, 24.54)	4.69 (0.94, 19.20)	0.790
White blood cell count, ×10⁹/L	13.50 (9.61, 19.28)	13.36 (9.47, 19.18)	14.46 (10.06, 19.56)	0.274
Neutrophil count, ×10⁹/L	12.07 (8.28, 17.32)	11.70 (7.94, 17.19)	13.23 (8.77, 17.74)	0.113
Lymphocyte count, ×10⁹/L	0.73 (0.43, 1.17)	0.73 (0.43, 1.19)	0.74 (0.42, 1.14)	0.847
Hemoglobin, g/L	99.00 (83.00, 117.00)	99.00 (83.00, 116.00)	100.00 (84.00, 117.00)	0.670
Platelet count, ×10⁹/L	169.00 (91.50, 228.00)	169.00 (96.00, 229.00)	169.50 (75.75, 224.50)	0.346
Total protein, g/L	49.93 ± 10.37	50.13 ± 10.65	49.48 ± 9.70	0.552
Albumin, g/L	25.95 ± 5.18	25.87 ± 5.30	26.14 ± 4.91	0.612
Total bilirubin, μmol/L	16.10 (10.60, 25.25)	16.00 (10.50, 24.50)	16.15 (10.93, 26.12)	0.606
Direct bilirubin, μmol/L	5.80 (3.10, 10.90)	5.50 (3.10, 10.90)	6.05 (3.52, 11.95)	0.164
ALT, U/L	25.20 (13.15, 54.80)	24.60 (12.90, 53.60)	25.85 (13.40, 61.12)	0.454
AST, U/L	39.40 (23.70, 88.10)	37.70 (22.80, 87.80)	44.50 (27.60, 90.93)	0.214
Urea, mmol/L	11.30 (7.60, 18.50)	11.70 (7.64, 17.90)	10.72 (7.43, 20.62)	0.958
Creatinine, μmol/L	97.00 (65.75, 161.30)	96.90 (64.50, 161.40)	97.20 (70.83, 153.23)	0.875
LDH, U/L	296.40 (217.35, 467.65)	288.80 (214.00, 461.00)	326.40 (218.48, 468.50)	0.280
Creatine kinase, U/L	127.50 (46.85, 360.75)	123.60 (46.10, 327.40)	132.75 (50.48, 428.48)	0.303
CK-MB, IU/L	16.70 (11.15, 28.80)	16.50 (10.90, 28.70)	17.10 (11.30, 29.15)	0.866
Lactate, mmol/L	2.80 (1.86, 5.12)	2.88 (1.86, 5.18)	2.62 (1.90, 4.82)	0.488
Oxygenation index, mmHg	172.35 (97.64, 229.58)	175.44 (101.05, 236.80)	156.55 (93.47, 212.77)	0.083
PT, s	13.70 (12.30, 16.50)	13.70 (12.30, 16.40)	13.55 (12.40, 16.98)	0.699
INR	1.19 (1.08, 1.45)	1.20 (1.07, 1.43)	1.19 (1.08, 1.51)	0.680
APTT, s	32.00 (28.20, 37.95)	31.70 (28.20, 37.40)	32.20 (28.05, 38.85)	0.876
TT, s	16.50 (15.50, 18.10)	16.60 (15.60, 18.10)	16.40 (15.40, 17.90)	0.228

SBP, systolic blood pressure; DBP, diastolic blood pressure; MAP, mean arterial pressure; SOFA, Sequential Organ Failure Assessment; APACHE II, Acute Physiology and Chronic Health Evaluation II; CRRT, continuous renal replacement therapy; ALT, alanine aminotransferase; AST, aspartate aminotransferase; LDH, lactate dehydrogenase; CK-MB, creatine kinase-MB isoenzyme; PT, prothrombin time; INR, international normalized ratio; APTT, activated partial thromboplastin time; TT, thrombin time.

### Feature selection

Feature selection considered 39 candidate predictors and was restricted to the training set to avoid information leakage. Boruta identified 12 confirmed predictors: lactate dehydrogenase, APACHE II score, urea, age, SOFA score, CRRT use, creatine kinase, thrombin time, INR, procalcitonin, prothrombin time, and vasoactive drug use. The Boruta feature selection results are shown in Figure 2.

**Figure 2:**
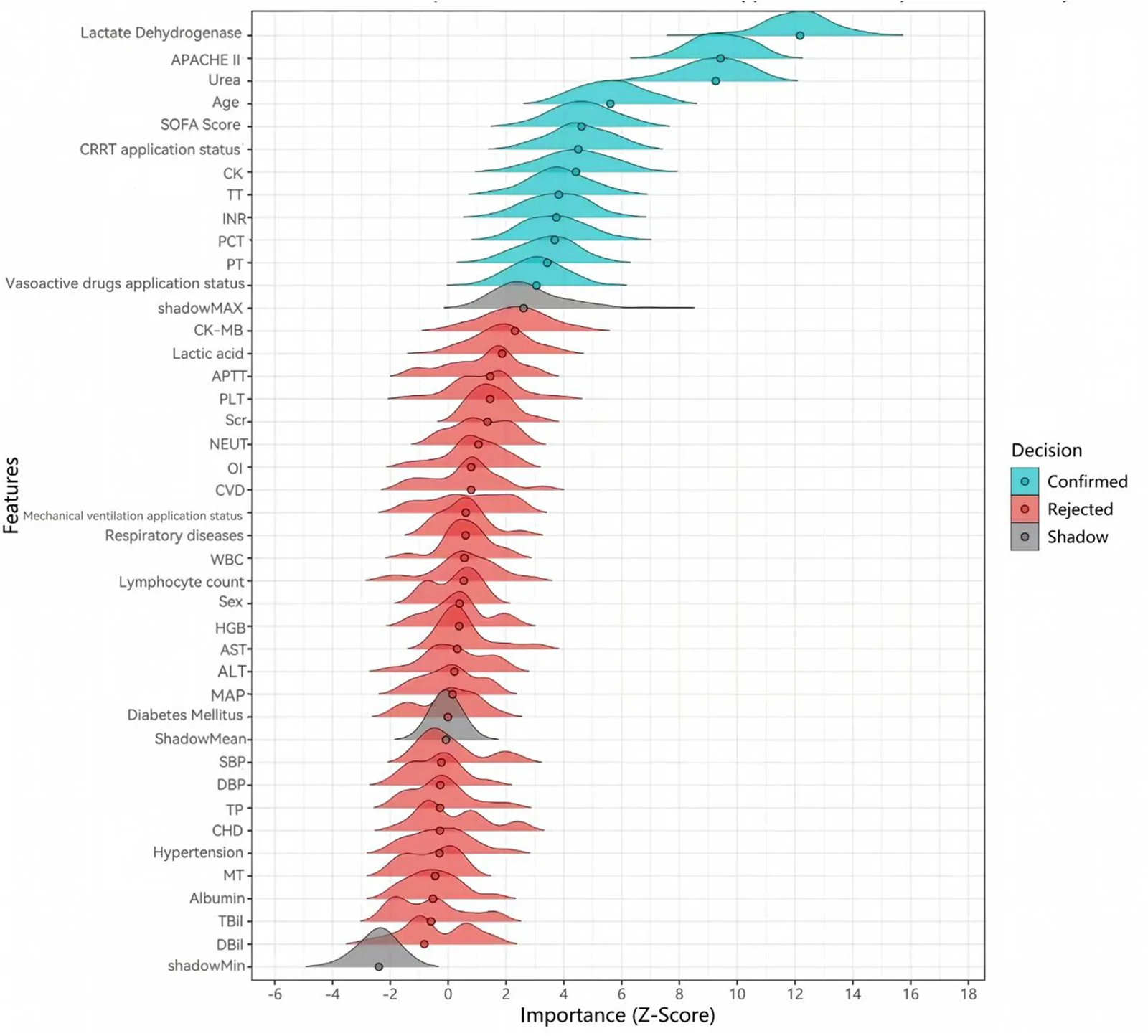
Feature selection using the boruta algorithm. Boruta feature selection was performed in the training set to identify predictors associated with in-hospital mortality. Confirmed variables were retained for subsequent machine learning model construction.

### Model performance and selection of the final model

Eight supervised machine learning models were developed and evaluated, including LR, NB, SVM, SHLNN, RF, LightGBM, KNN and DT. Model performance was assessed in both the training and validation sets using discrimination, classification, calibration, and clinical utility measures. Table 3 presents the classification and calibration performance of all models. In the validation set, RF showed the best overall balance, achieving an AUC of 0.804 (95% CI, 0.730–0.879), sensitivity of 0.817, specificity of 0.757, positive predictive value of 0.742, negative predictive value of 0.828, accuracy of 0.785, F1 score of 0.778, Brier score of 0.1858, and expected calibration error of 0.1082. Although LightGBM achieved the highest sensitivity and KNN had the lowest ECE, RF provided the most balanced overall performance across discrimination, sensitivity and specificity, classification accuracy, calibration, and clinical utility. Therefore, RF was selected as the final model based on its overall performance profile rather than superiority in any single metric.

**Table 3:** Performance comparison of machine learning models in the training and validation sets.

Model	AUC (95% CI)	Sensitivity	Specificity	PPV	NPV	Accuracy	F1 score	Brier score	ECE
Training set									
LR	0.708 (0.649–0.766)	0.833	0.521	0.596	0.787	0.664	0.695	0.2189	0.1128
NB	0.726 (0.668–0.783)	0.609	0.767	0.689	0.698	0.694	0.646	0.2399	0.1871
SVM	0.710 (0.652–0.767)	0.906	0.436	0.576	0.845	0.651	0.704	0.2172	0.0915
SHLNN	0.707 (0.649–0.766)	0.862	0.515	0.601	0.816	0.674	0.708	0.2197	0.1066
RF	0.761 (0.707–0.815)	0.761	0.656	0.652	0.764	0.704	0.702	0.1985	0.0413
LightGBM	0.755 (0.701–0.810)	0.768	0.656	0.654	0.770	0.708	0.707	0.1999	0.0361
KNN	0.668 (0.607–0.728)	0.790	0.485	0.565	0.731	0.625	0.659	0.2352	0.1068
DT	0.623 (0.560–0.686)	0.536	0.675	0.583	0.632	0.611	0.558	0.2440	0.1112
Validation set									
LR	0.775 (0.695–0.855)	0.733	0.714	0.688	0.758	0.723	0.710	0.1914	0.0569
NB	0.679 (0.586–0.773)	0.800	0.514	0.585	0.750	0.646	0.676	0.2699	0.2294
SVM	0.780 (0.701–0.859)	0.700	0.800	0.750	0.757	0.754	0.724	0.1898	0.0855
SHLNN	0.736 (0.650–0.819)	0.783	0.600	0.627	0.764	0.685	0.696	0.2163	0.1807
RF	0.804 (0.730–0.879)	0.817	0.757	0.742	0.828	0.785	0.778	0.1858	0.1082
LightGBM	0.800 (0.726–0.875)	0.850	0.643	0.671	0.833	0.738	0.750	0.1864	0.1198
KNN	0.755 (0.674–0.837)	0.600	0.771	0.692	0.692	0.692	0.643	0.1991	0.0538
DT	0.555 (0.463–0.648)	0.800	0.329	0.505	0.657	0.546	0.619	0.2695	0.1648

LR, logistic regression; NB, naive Bayes; SVM, support vector machine; SHLNN, single-layer hidden-layer neural network; RF, random forest; LightGBM, Light Gradient Boosting Machine; KNN, K-nearest neighbors; DT, decision tree; AUC, area under the receiver operating characteristic curve; CI, confidence interval; PPV, positive predictive value; NPV, negative predictive value; ECE, expected calibration error.

ROC curves, calibration plots, and decision curve analysis are presented in Figure 3. The RF model showed acceptable discrimination in the validation set, with predicted probabilities generally aligning with observed outcomes. Decision curve analysis indicated potential clinical net benefit across relevant threshold probabilities. These results supported the choice of RF for model interpretation and deployment.

**Figure 3:**
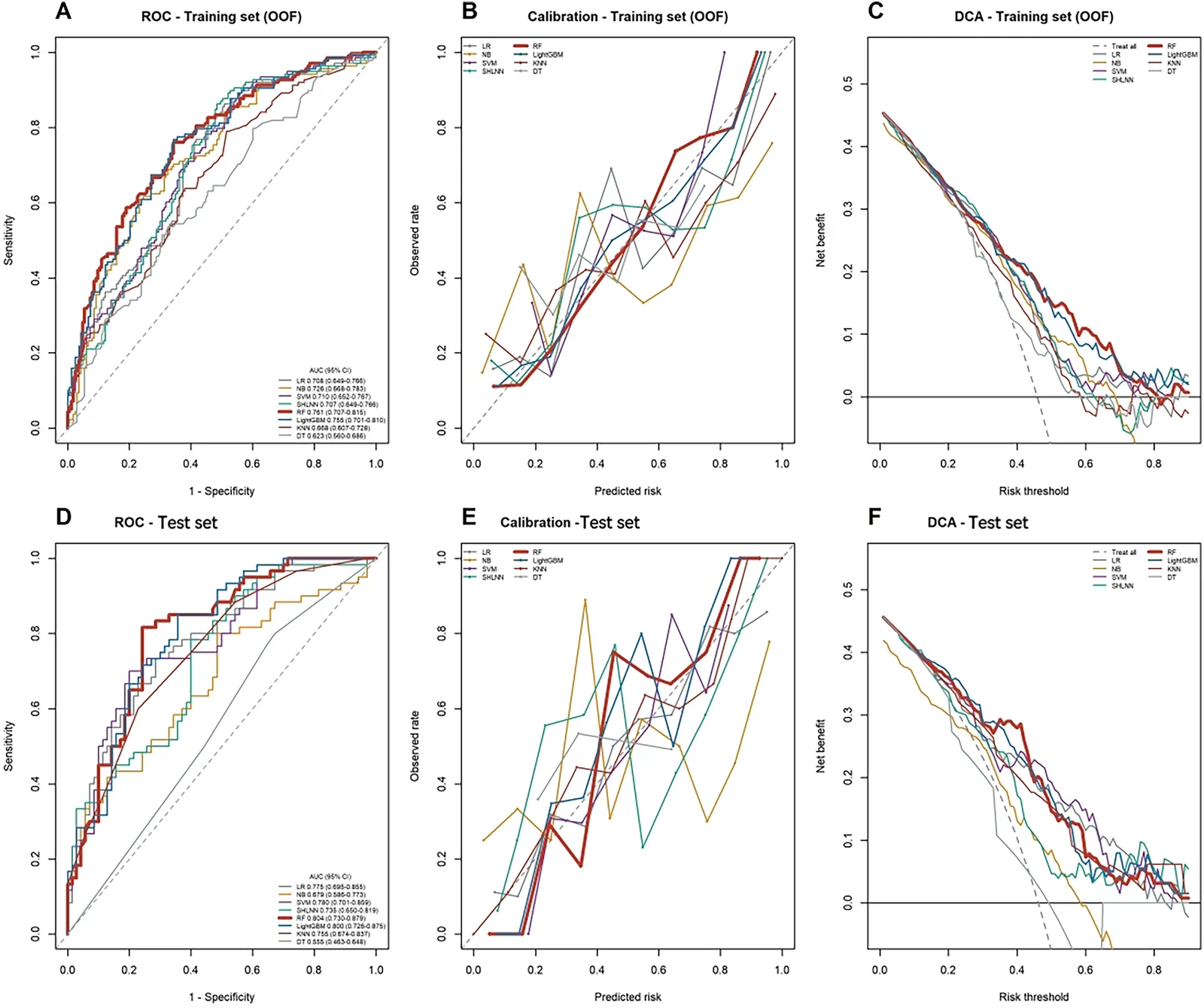
Performance comparison of machine learning models. ROC curves, calibration curves, and decision curve analysis were used to compare the performance of eight machine learning models in the training and validation sets **(A)** ROC curves for the out-of-fold (OOF) predictions in the training set. **(B)** Calibration curves for the OOF predictions in the training set. **(C)** Decision curve analysis (DCA) for the OOF predictions in the training set. **(D)** ROC curves for the independent test set. **(E)** Calibration curves for the independent test set. **(F)** DCA for the independent test set. ROC, receiver operating characteristic; DCA, decision curve analysis; RF, random forest.

To determine whether the final RF model provided additional discriminative value compared with established clinical severity scores, we further compared its performance with the SOFA and APACHE II scores in the validation set. The RF model achieved an AUC of 0.804 (95% CI, 0.730–0.879), compared with 0.613 (95% CI, 0.514–0.711) for SOFA and 0.690 (95% CI, 0.598–0.782) for APACHE II. Pairwise DeLong tests showed that the AUC of the RF model was significantly higher than that of SOFA (*P* < 0.001) and APACHE II (*P* = 0.017). The detailed comparisons are presented in Supplementary Table S3, and the corresponding ROC curves are shown in Supplementary Figure S1.

### SHAP-based model interpretation

SHAP analysis was performed to interpret the final RF model. The global SHAP summary plot and feature importance plot are shown in Figure 4. Lactate dehydrogenase showed the highest mean absolute SHAP value, followed by urea, age, APACHE II score, procalcitonin, creatine kinase, thrombin time, SOFA score, prothrombin time, INR, CRRT use, and vasoactive drug use. These findings indicate that model predictions were primarily driven by markers of tissue injury and renal dysfunction, together with age, overall illness severity, infection-related inflammatory status, coagulation abnormalities, and organ-support requirements.

**Figure 4:**
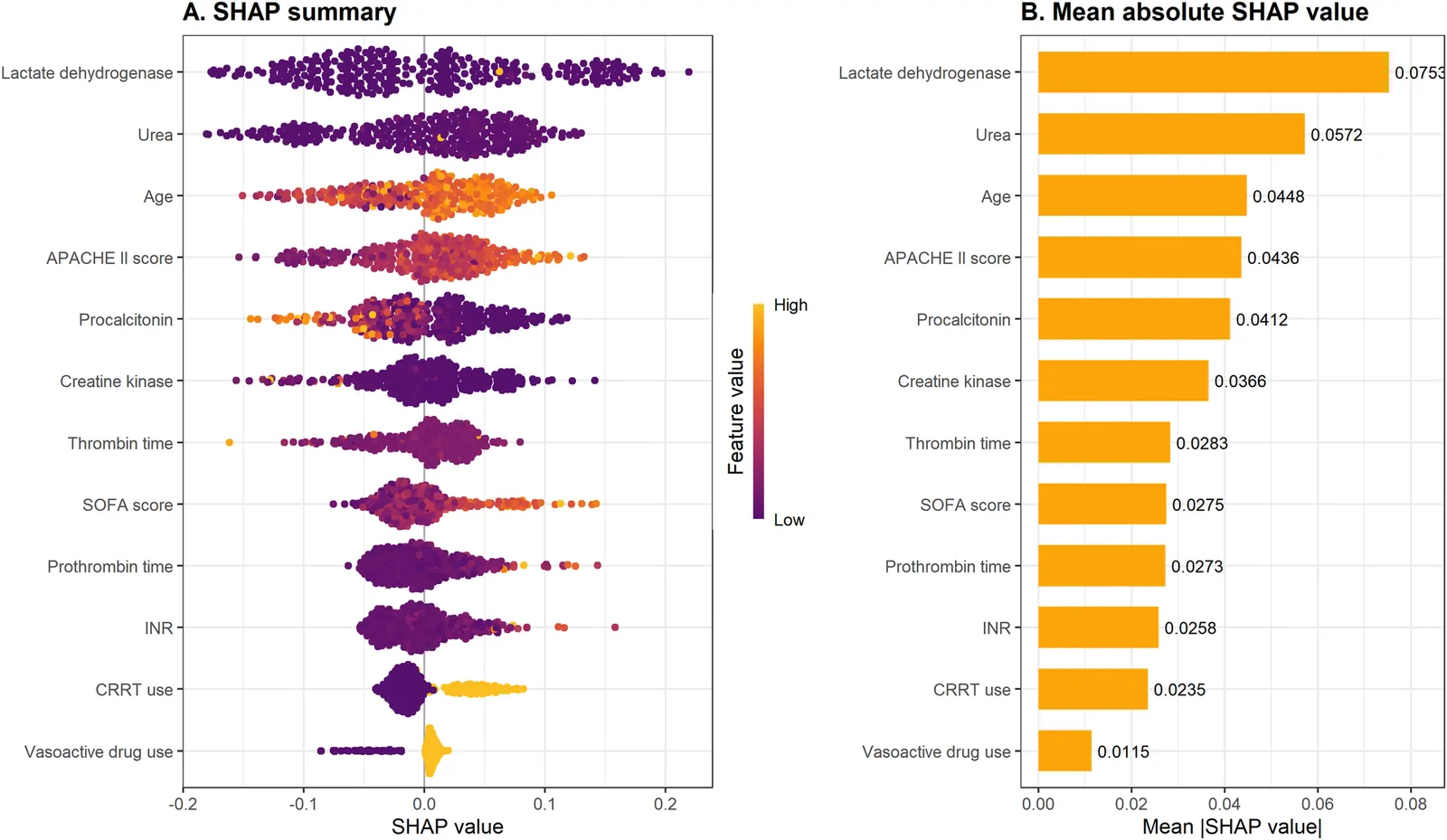
Global SHAP interpretation of the final random forest model. **(A)** SHAP summary plot showing the contribution of each predictor to the predicted risk of in-hospital mortality. Each dot represents one patient; positive SHAP values indicate higher predicted risk and negative values indicate lower predicted risk. Color represents the feature value from low to high. **(B)** SHAP feature importance plot ranking predictors by mean absolute SHAP value, with larger values indicating greater overall contribution to model predictions.

Individual-level SHAP waterfall plots are shown in Figure 5. These plots illustrated how different predictors contributed to the predicted probability of in-hospital mortality in representative patients. Some predictors increased the predicted risk, whereas others decreased it, demonstrating that the final model could provide individualized risk explanations rather than only a binary classification result. SHAP dependence plots further illustrated the relationships between selected predictors and their contributions to model output, suggesting that several predictors had nonlinear associations with predicted mortality risk (Figure 6).

**Figure 5:**
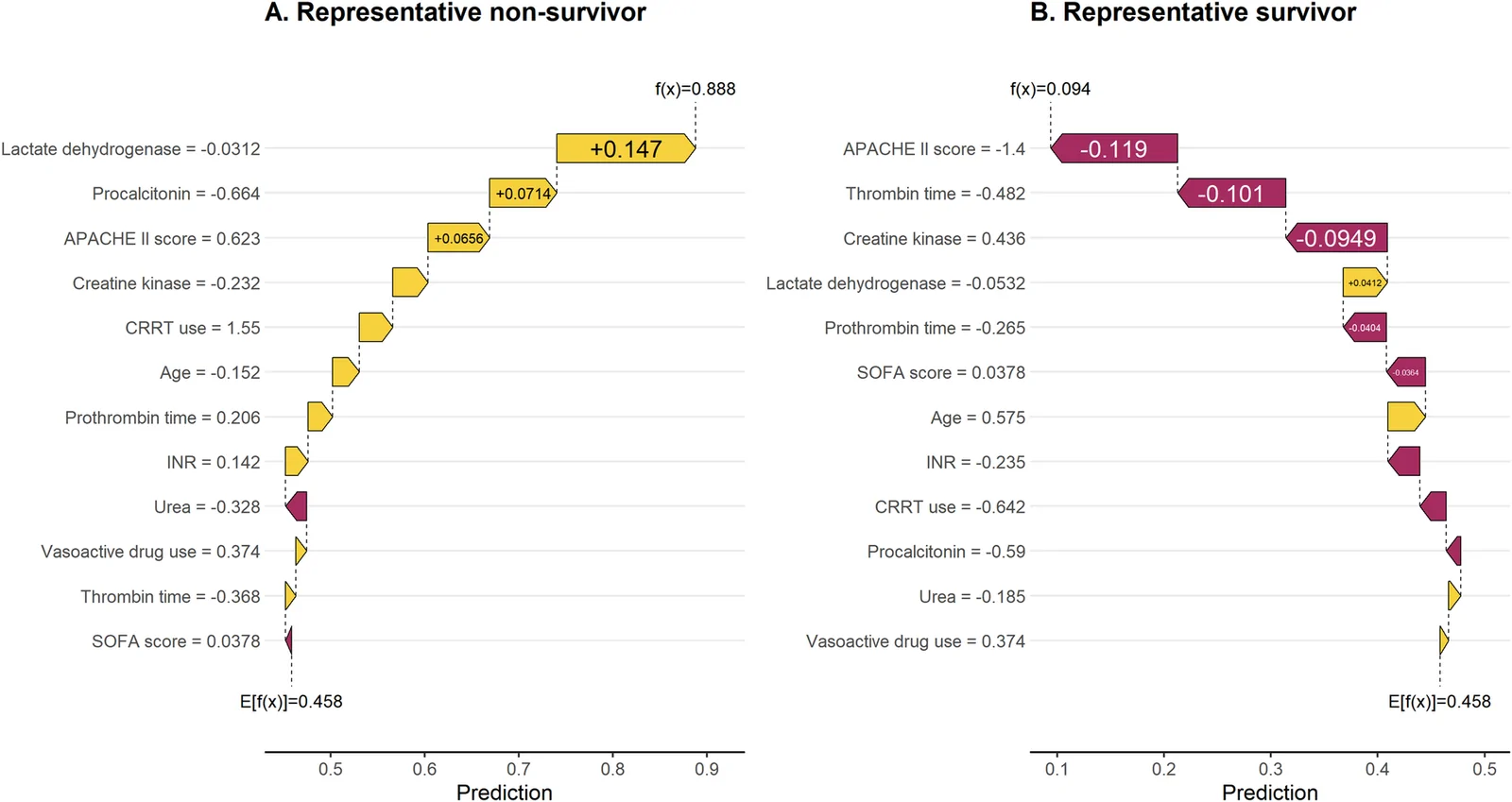
Individual-level SHAP explanations of the final random forest model. SHAP waterfall plots showing how individual predictors contributed to the model prediction for representative patients. Positive contributions increase the predicted risk of in-hospital mortality, whereas negative contributions decrease it. Panels A and B show SHAP waterfall plots for a representative non-survivor and survivor, respectively. E[f(x)] = 0.458 represents the model's baseline prediction, namely the mean predicted mortality risk in the reference population. f(x) represents the final predicted mortality risk for an individual patient: 0.888 for the non-survivor and 0.094 for the survivor. The horizontal axis labeled “Prediction” indicates the model-predicted mortality risk. Yellow arrows represent features that increase the predicted mortality risk, whereas magenta arrows represent features that decrease it. The numbers displayed inside the arrows are SHAP values, with their signs indicating the direction of the effect and their absolute magnitudes indicating the strength of each feature's contribution. The values following the equals signs beside the feature names are the corresponding model input values for the individual patient and are standardized values rather than original clinical measurements.

**Figure 6:**
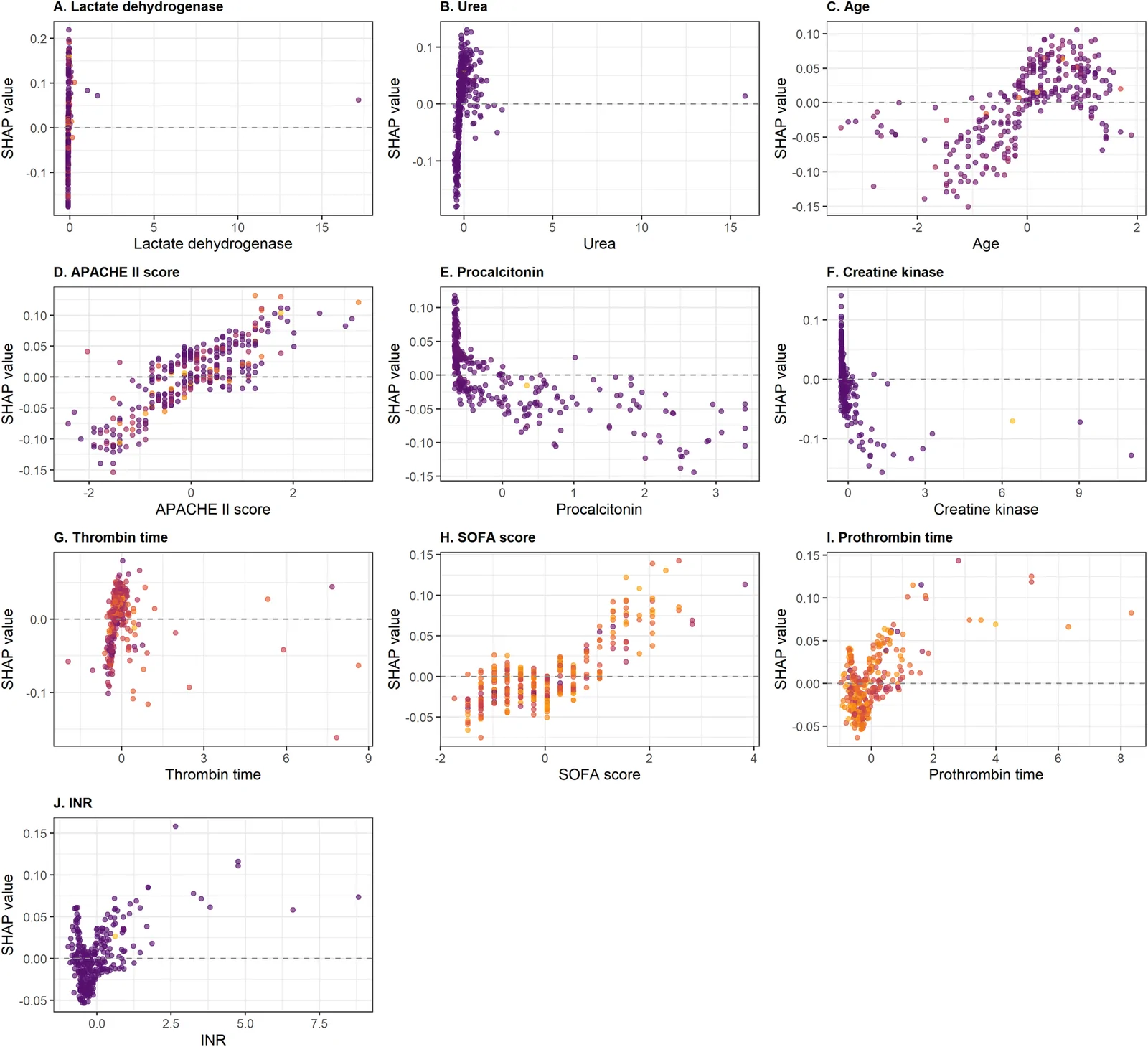
SHAP dependence plots of key predictors. Each dot represents one patient. The *x*-axis shows the predictor value and the *y*-axis shows the corresponding SHAP value, illustrating how each predictor contributes to the predicted risk of in-hospital mortality across its range. Panels A–J show SHAP dependence plots for lactate dehydrogenase, urea, age, APACHE II score, procalcitonin, creatine kinase, thrombin time, SOFA score, prothrombin time, and INR, respectively. Each dot represents an individual patient. The horizontal axis shows the value of the corresponding feature used by the prediction model, whereas the vertical axis shows its SHAP value. A positive SHAP value indicates that the feature increases the model-predicted mortality risk, while a negative SHAP value indicates that it decreases the predicted risk. The horizontal dashed line at a SHAP value of zero represents no contribution to the prediction. The farther a point lies from zero, the greater the influence of that feature on the individual prediction.

### Web-based calculator

To demonstrate the practical deployment of the final random forest model, we developed an interactive web-based calculator using Streamlit. The interface of the calculator is shown in Figure 7. Users can input the selected predictors and obtain the corresponding estimated probability of in-hospital mortality. The web application is available at: https://clinical-rf-demo-m9nkujyucv6t9ewgykzzbl.streamlit.app/.

**Figure 7:**
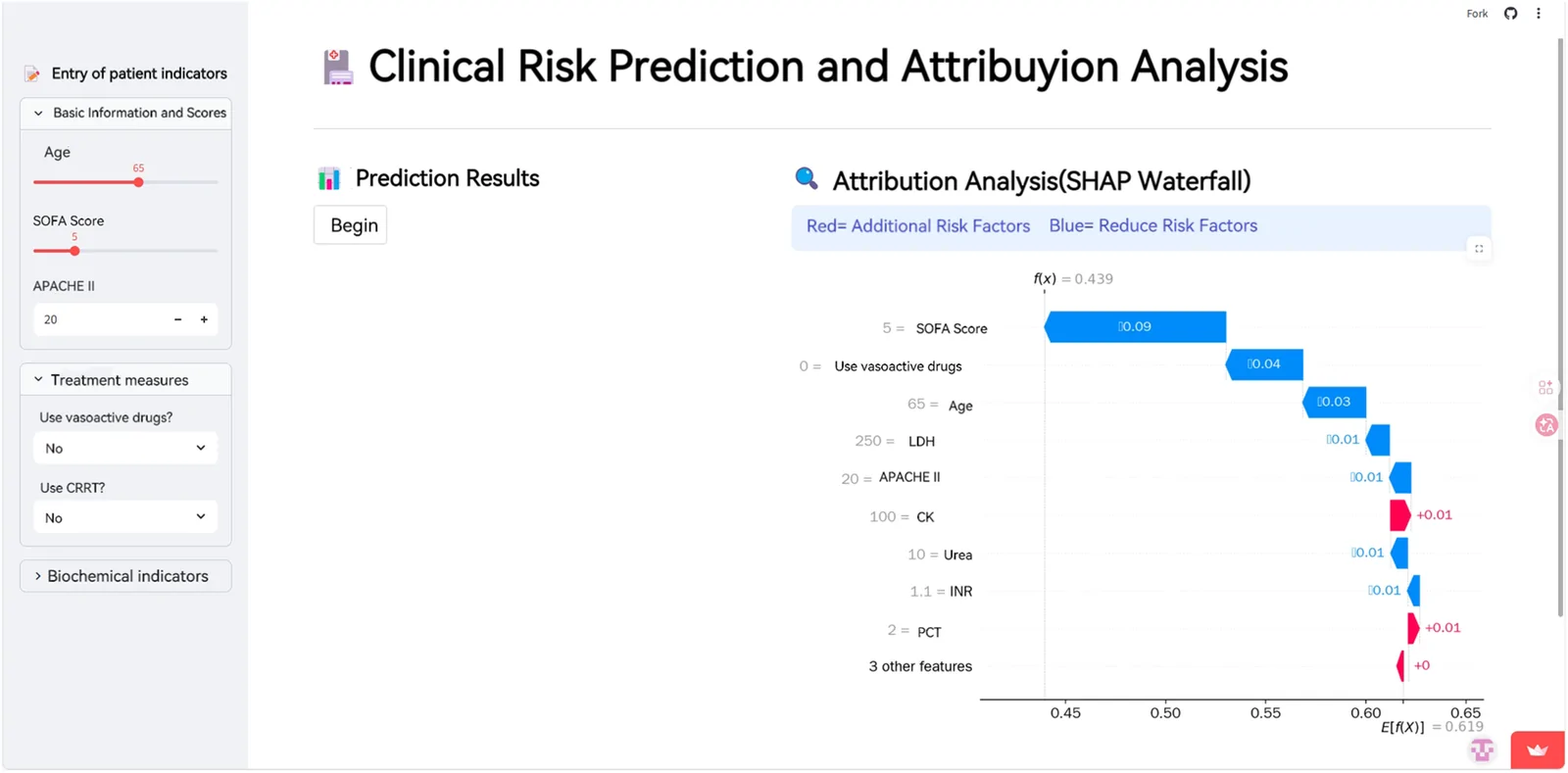
Web-based calculator for mortality risk prediction. The final random forest model was deployed as an interactive web-based calculator to estimate the individualized probability of in-hospital mortality in patients with sepsis.

## Discussion

In this retrospective cohort of patients with sepsis admitted to the ICU at Hebei Provincial People's Hospital, we constructed and internally validated machine learning models to estimate the risk of in-hospital death using routinely collected clinical variables. Among 431 eligible patients, 198 died during hospitalization, indicating a high-risk critically ill population. Following random partitioning, 301 patients formed the training cohort and 130 formed the validation cohort. Among the eight machine learning algorithms assessed, RF provided the best balance of validation performance, yielding a sensitivity of 0.817, specificity of 0.757, accuracy of 0.785, F1 score of 0.778, Brier score of 0.1858, and expected calibration error of 0.1082. Importantly, the present study did not merely construct a prediction model, but further used Boruta-based feature selection and SHAP-based interpretation to translate model outputs into clinically understandable risk patterns. These findings suggest that an interpretable machine learning framework based on early ICU variables may support individualized mortality risk stratification in patients with sepsis.

Sepsis, defined as life-threatening organ dysfunction resulting from a dysregulated host response to infection, remains a major contributor to global mortality despite advances in critical care (1, 2). The Surviving Sepsis Campaign highlights the importance of timely diagnosis, early initiation of antimicrobial treatment, effective source control, and appropriate hemodynamic resuscitation (18). However, early prognostic assessment remains challenging because sepsis is highly heterogeneous, and mortality risk is shaped by the interaction of infection burden, host vulnerability, organ dysfunction, hemodynamic instability, coagulation disorder, and treatment response. Traditional severity scores such as SOFA and APACHE II have been widely used to quantify organ dysfunction and physiological derangement (6, 7). Nevertheless, these scoring systems are based on predefined variables and weighting schemes, and may inadequately reflect nonlinear associations and higher-order interactions between clinical variables. Therefore, machine learning models may provide complementary value by integrating multidimensional information into individualized risk estimates.

Several studies have investigated the use of machine learning for predicting mortality in patients with sepsis. Hu and colleagues developed and validated interpretable models for the early estimation of in-hospital death among critically ill patients with sepsis, demonstrating that SHAP analysis can enhance model transparency (14). In postoperative sepsis, Yao and colleagues reported superior in-hospital mortality prediction with XGBoost compared with stepwise logistic regression and a baseline model, with coagulopathy, renal replacement therapy, urine output, and fluid–electrolyte imbalance ranking among the most influential predictors (19). Park et al. used nationwide multicenter data from Korea and found that machine learning models based on baseline clinical variables achieved better mortality prediction than SOFA components alone (15). Zeng et al. further showed that a blending machine learning model based on ICU sepsis patients outperformed SAPS II and SOFA in external validation using two large ICU databases (20). Compared with these studies, our work was based on a real-world ICU cohort from a Chinese tertiary hospital and focused on routinely available variables collected early during ICU care. Although the sample size was smaller than those of large public-database studies, this local cohort may better reflect institution-specific case mix, treatment patterns, and data structure, which is important for model implementation in real clinical settings.

The selection of RF was based on overall performance rather than superiority in any single metric. Although differences among several competing models were modest, RF provided a favorable balance of discrimination, sensitivity, specificity, accuracy, F1 score, calibration, and clinical net benefit in the validation set. Although LightGBM achieved a slightly higher sensitivity and some competing models performed better on individual metrics, RF showed the most balanced overall performance profile. Therefore, RF was selected as the final model as a pragmatic choice based on its overall performance rather than as evidence of universal superiority over the other algorithms. The RF model also showed significantly better discrimination than SOFA or APACHE II alone in the validation set. This suggests that combining established severity scores with additional routinely available clinical variables may improve prognostic discrimination. However, external validation is still required before clinical superiority can be established.

The SHAP analysis provided further insight into the variables contributing to the RF model predictions. Lactate dehydrogenase and urea showed the highest global SHAP importance, while age was also among the leading predictors. Lactate dehydrogenase is a nonspecific marker of cellular injury and may reflect tissue hypoperfusion, metabolic stress, and multiorgan dysfunction; previous studies have also reported an association between elevated Lactate dehydrogenase and mortality in patients with sepsis (21). Although our model included serum urea rather than blood urea nitrogen (BUN), the prominence of this renal-related marker is consistent with previous evidence showing that BUN is independently associated with mortality in critically ill patients with sepsis (22). Age may further reflect differences in physiological reserve and host responses to severe infection, which may contribute to poorer outcomes in older patients with sepsis (23, 24).

SOFA and APACHE II also contributed to the model, supporting the importance of organ dysfunction and overall physiological severity in mortality risk assessment. Although some individual predictors overlap with clinical domains represented in SOFA and APACHE II, they may provide complementary information. Composite scores summarize physiological abnormalities using predefined thresholds, whereas individual continuous variables may retain more granular information and capture nonlinear patterns or interactions beyond these scoring categories. Random forest models can accommodate correlated predictors; however, overlapping variables may share or redistribute feature importance. Therefore, SHAP rankings should not be interpreted as independent or causal effects of individual predictors.

Among infection-related variables, procalcitonin was included as an informative predictor. Procalcitonin has been extensively evaluated as a biomarker for bacterial infection and sepsis, although its prognostic implications remain complex (25, 26). Unexpectedly, baseline PCT levels were higher in survivors than in non-survivors. This finding should be interpreted cautiously and should not be taken to indicate a protective effect of higher PCT. Several factors may partly explain this pattern, including differences in the timing of PCT measurement, infection etiology, prior antimicrobial exposure, immune responsiveness, and the stage of sepsis at ICU admission. In addition, the between-group comparison represents an unadjusted association, whereas Boruta selection and SHAP importance indicate only that PCT contributed predictive information within the multivariable model, potentially through nonlinear relationships and interactions with other variables. Because detailed microbiological characteristics and comprehensive immunosuppressive status were unavailable, these explanations could not be directly examined. Future studies incorporating infection etiology, immune status, and serial PCT measurements are needed to clarify this unexpected finding.

Other predictors, including creatine kinase, coagulation parameters, CRRT, and vasoactive drug use, provided additional information on the severity and systemic consequences of sepsis. Creatine kinase may reflect tissue injury, whereas abnormalities in TT, PT, and INR are consistent with the coagulation disturbances frequently observed in sepsis (27, 28). CRRT and vasoactive drug use should be interpreted primarily as indicators of severe renal dysfunction, hemodynamic instability, and the requirement for advanced organ support rather than as causal determinants of mortality. Thus, the SHAP findings collectively suggest that mortality prediction in sepsis reflects information across tissue injury, renal and coagulation dysfunction, physiological severity, inflammatory status, and organ-support requirements, rather than being driven by any single clinical factor.

The potential clinical value of this study lies in providing an interpretable framework for early risk stratification. If externally and prospectively validated, the web-based model may assist clinicians in identifying patients at higher estimated risk of in-hospital mortality. At present, however, the calculator should be regarded as a research prototype rather than a tool for routine clinical decision-making. Its clinical utility and impact on patient management require further evaluation in independent cohorts and prospective studies.

Several limitations warrant consideration. First, this was a single-center retrospective study conducted at Hebei Provincial People's Hospital, and the model may therefore reflect local patient characteristics, ICU admission criteria, treatment practices, and documentation patterns. Second, validation was limited to a random internal train-validation split. Although this approach provides an initial estimate of model performance, it does not establish transportability to other institutions or patient populations. Third, the total sample size was modest, with 431 patients and only 130 patients in the validation set, which may increase the uncertainty of performance estimates and limit model stability. Independent external validation in larger, multicenter cohorts is therefore required before the model can be considered for routine clinical use. Fourth, the model was based on variables collected during the early ICU period and did not incorporate dynamic trajectories, such as changes in lactate, SOFA score, vasopressor dose, renal function, oxygenation, or inflammatory biomarkers over time. Previous studies have suggested that dynamic vital sign patterns and longitudinal data may further improve prognostic prediction in sepsis (29). In addition, detailed microbiological characteristics and comprehensive information on immunosuppressive status were not consistently available, limiting our ability to further investigate the unexpected distribution of baseline PCT between survivors and non-survivors. Fifth, treatment-related variables, including vasoactive drug administration, mechanical ventilation, and CRRT, may primarily reflect underlying illness severity and clinician decision-making rather than directly modifiable causal determinants. Finally, SHAP analysis improves transparency by explaining the contribution of variables to model output, but it does not establish causality. Prospective validation, external testing, and clinical impact studies are required before this model can be recommended for routine bedside use.

In conclusion, this study developed an interpretable machine learning model for predicting in-hospital mortality in ICU patients with sepsis using routinely available clinical variables. The model showed acceptable discrimination and calibration, while SHAP analysis improved model interpretability. The accompanying web-based calculator serves as a research prototype demonstrating the feasibility of model deployment rather than an established clinical decision-support tool. Given the single-center design, modest sample size, and lack of external validation, these findings should be considered preliminary. Further multicenter and prospective validation is required before clinical application.

## Data Availability

The data analyzed in this study is subject to the following licenses/restrictions: The datasets generated and analyzed during this study are not publicly available because of patient privacy and institutional data governance requirements. Requests for data access should be directed to the corresponding author and will require approval from Hebei Provincial People's Hospital and the relevant ethics committee. Requests to access these datasets should be directed to Name:Tianyu Zhao. Email:abc774187661@qq.com.

## References

[1] Singer M Deutschman CS Seymour CW Shankar-Hari M Annane D Bauer M. The third international consensus definitions for sepsis and septic shock (sepsis-3). Jama. (2016) 315(8):801–10. 10.1001/jama.2016.0287 26903338 PMC4968574

[2] Rudd KE Johnson SC Agesa KM Shackelford KA Tsoi D Kievlan DR. Global, regional, and national sepsis incidence and mortality, 1990–2017: analysis for the global burden of disease study. Lancet. (2020) 395(10219):200–11. 10.1016/s0140-6736(19)32989-7 31954465 PMC6970225

[3] Sakr Y Jaschinski U Wittebole X Szakmany T Lipman J Ñamendys-Silva SA. Sepsis in intensive care unit patients: worldwide data from the intensive care over nations audit. Open Forum Infect Dis. (2018) 5(12):ofy313. 10.1093/ofid/ofy313 30555852 PMC6289022

[4] Seymour CW Liu VX Iwashyna TJ Brunkhorst FM Rea TD Scherag A. Assessment of clinical criteria for sepsis: for the third international consensus definitions for sepsis and septic shock (sepsis-3). Jama. (2016) 315(8):762–74. 10.1001/jama.2016.0288 26903335 PMC5433435

[5] Hotchkiss RS Monneret G Payen D. Sepsis-Induced immunosuppression: from cellular dysfunctions to immunotherapy. Nat Rev Immunol. (2013) 13(12):862–74. 10.1038/nri3552 24232462 PMC4077177

[6] Vincent J-L De Mendonca A Cantraine F Moreno R Takala J Suter PM. Use of the Sofa score to assess the incidence of organ dysfunction/failure in intensive care units: results of a multicenter, prospective study. Working group on “sepsis-related problems” of the European society of intensive care medicine. Crit Care Med. (1998) 26(11):1793–800. 10.1097/00003246-199811000-00016 9824069

[7] Knaus WA Draper EA Wagner DP Zimmerman JE. Apache ii: a severity of disease classification system. Crit Care Med. (1985) 13(10):818–29. 10.1097/00003246-198510000-00009 3928249

[8] Le Gall JR Lemeshow S Saulnier F. A new simplified acute physiology score (saps ii) based on a European/north American multicenter study. Jama. (1993) 270(24):2957–63. 10.1001/jama.270.24.2957 8254858

[9] Baig MA Sheikh S Hussain E Bakhtawar S Subhan Khan M Mujtaba S. Comparison of qsofa and Sofa score for predicting mortality in severe sepsis and septic shock patients in the emergency department of a low middle income country. Turk J Emerg Med. (2018) 18(4):148–51. 10.1016/j.tjem.2018.08.002 30533557 PMC6261097

[10] Papapanagiotou I Karalis A Kokkoris S Vrettou CS Kampouropoulou O Giannopoulou V. Machine learning for early detection and prediction of sepsis: explainability and key sepsis biomarkers representation-a systematic review. Int J Med Inform. (2026) 214:106420. 10.1016/j.ijmedinf.2026.106420 41962401

[11] Nikravangolsefid N Reddy S Truong HH Charkviani M Ninan J Prokop LJ. Machine learning for predicting mortality in adult critically ill patients with sepsis: a systematic review. J Crit Care. (2024) 84:154889. 10.1016/j.jcrc.2024.154889 39059094

[12] Lundberg SM Lee SI. A unified approach to interpreting model predictions. In: Proceedings of the 31st International Conference on Neural Information Processing Systems (Long Beach, California, USA: Curran Associates Inc) (2017), 4768–77

[13] Ribeiro MT Singh S Guestrin C. “Why should I trust you?”: explaining the predictions of any classifier. In: Proceedings of the 22nd ACM SIGKDD International Conference on Knowledge Discovery and Data Mining) (2016).

[14] Hu C Li L Huang W Wu T Xu Q Liu J. Interpretable machine learning for early prediction of prognosis in sepsis: a discovery and validation study. Infect Dis Ther. (2022) 11(3):1117–32. 10.1007/s40121-022-00628-6 35399146 PMC9124279

[15] Park SW Yeo NY Kang S Ha T Kim T-H Lee DH. Early prediction of mortality for septic patients visiting emergency room based on explainable machine learning: a real-world multicenter study. J Korean Med Sci. (2024) 39(5):e53. 10.3346/jkms.2024.39.e53 38317451 PMC10843974

[16] Wang M Jiang L Zhu B Li W Du B Kang Y. The prevalence, risk factors, and outcomes of sepsis in critically ill patients in China: a multicenter prospective cohort study. Front Med. (2020) 7:593808. 10.3389/fmed.2020.593808 PMC7774866 33392219

[17] Zhou M Wang H Zeng X Yin P Zhu J Chen W. Mortality, morbidity, and risk factors in China and its provinces, 1990–2017: a systematic analysis for the global burden of disease study 2017. Lancet. (2019) 394(10204):1145–58. 10.1016/s0140-6736(19)30427-1 31248666 PMC6891889

[18] Evans L Rhodes A Alhazzani W Antonelli M Coopersmith CM French C. Surviving sepsis campaign: international guidelines for management of sepsis and septic shock 2021. Crit Care Med. (2021) 49(11):e1063–e143. 10.1097/ccm.0000000000005337 34605781

[19] Yao R Jin X Wang G Yu Y Wu G Zhu Y. A machine learning-based prediction of hospital mortality in patients with postoperative sepsis. Front Med. (2020) 7:445. 10.3389/fmed.2020.00445 PMC7438711 32903618

[20] Zeng Z Yao S Zheng J Gong X. Development and validation of a novel blending machine learning model for hospital mortality prediction in icu patients with sepsis. BioData Min. (2021) 14(1):40. 10.1186/s13040-021-00276-5 34399809 PMC8365981

[21] Lu J Wei Z Jiang H Cheng L Chen Q Chen M. Lactate dehydrogenase is associated with 28-day mortality in patients with sepsis: a retrospective observational study. J Surg Res. (2018) 228:314–21. 10.1016/j.jss.2018.03.035 29907227

[22] Harazim M Tan K Nalos M Matejovic M. Blood urea nitrogen—independent marker of mortality in sepsis. Biomed Pap Med Fac Univ Palacky Olomouc Czech Repub. (2023) 167(1):24–9. 10.5507/bp.2022.015 35373784

[23] Martín S Pérez A Aldecoa C. Sepsis and immunosenescence in the elderly patient: a review. Front Med (Lausanne). (2017) 4:20. 10.3389/fmed.2017.00020 PMC5329014 28293557

[24] Michels EHA Butler JM Reijnders TDY Cremer OL Scicluna BP Uhel F. Association between age and the host response in critically ill patients with sepsis. Crit Care. (2022) 26(1):385. 10.1186/s13054-022-04266-9 36514130 PMC9747080

[25] Wacker C Prkno A Brunkhorst FM Schlattmann P. Procalcitonin as a diagnostic marker for sepsis: a systematic review and meta-analysis. Lancet Infect Dis. (2013) 13(5):426–35. 10.1016/s1473-3099(12)70323-7 23375419

[26] Liu D Su L Han G Yan P Xie L. Prognostic value of procalcitonin in adult patients with sepsis: a systematic review and meta-analysis. PLoS One. (2015) 10(6):e0129450. 10.1371/journal.pone.0129450 26076027 PMC4468164

[27] Levi M Van Der Poll T. Coagulation and sepsis. Thromb Res. (2017) 149:38–44. 10.1016/j.thromres.2016.11.007 27886531

[28] Iba T Levy JH. Sepsis-Induced coagulopathy and disseminated intravascular coagulation. Anesthesiology. (2020) 132(5):1238–45. 10.1097/aln.0000000000003122 32044801

[29] Cheng CY Kung CT Chen FC Chiu IM Lin CHR Chu CC. Machine learning models for predicting in-hospital mortality in patient with sepsis: analysis of vital sign dynamics. Front Med. (2022) 9:964667. 10.3389/fmed.2022.964667 PMC9631306 36341257

