# Supplementary material for: Development of an interpretable machine learning model for predicting in-hospital mortality in ICU patients with sepsis: a retrospective cohort study
Source: Front Med (Lausanne). 2026 Sep 15;13:1889209. doi: 10.3389/fmed.2026.1889209 (PMC13619919; doi:10.3389/fmed.2026.1889209)
Supplement: Supplementary file 1 [file Supplementaryfile1.docx]

Supplementary Table 1. Missingness of candidate predictors before imputation

| **Variable** | **N** | **Missing, n** | **Missing, %** |
| --- | --- | --- | --- |
| Procalcitonin | 431 | 22 | 5.10 |
| Lactate | 431 | 7 | 1.62 |
| Age | 431 | 3 | 0.70 |
| ALT | 431 | 1 | 0.23 |
| Creatinine | 431 | 1 | 0.23 |
| APACHE II score | 431 | 0 | 0.00 |
| APTT | 431 | 0 | 0.00 |
| AST | 431 | 0 | 0.00 |
| Albumin | 431 | 0 | 0.00 |
| CK-MB isoenzyme | 431 | 0 | 0.00 |
| CRRT use | 431 | 0 | 0.00 |
| Cerebrovascular disease | 431 | 0 | 0.00 |
| Coronary heart disease | 431 | 0 | 0.00 |
| Creatine kinase | 431 | 0 | 0.00 |
| Diabetes mellitus | 431 | 0 | 0.00 |
| Diastolic blood pressure | 431 | 0 | 0.00 |
| Direct bilirubin | 431 | 0 | 0.00 |
| Hemoglobin | 431 | 0 | 0.00 |
| Hypertension | 431 | 0 | 0.00 |
| INR | 431 | 0 | 0.00 |
| Lactate dehydrogenase | 431 | 0 | 0.00 |
| Lymphocyte count | 431 | 0 | 0.00 |
| Malignant tumor | 431 | 0 | 0.00 |
| Mean arterial pressure | 431 | 0 | 0.00 |
| Mechanical ventilation | 431 | 0 | 0.00 |
| Neutrophil count | 431 | 0 | 0.00 |
| Oxygenation index | 431 | 0 | 0.00 |
| PT | 431 | 0 | 0.00 |
| Platelet count | 431 | 0 | 0.00 |
| Respiratory disease | 431 | 0 | 0.00 |
| SOFA score | 431 | 0 | 0.00 |
| Sex | 431 | 0 | 0.00 |
| Systolic blood pressure | 431 | 0 | 0.00 |
| TT | 431 | 0 | 0.00 |
| Total bilirubin | 431 | 0 | 0.00 |
| Total protein | 431 | 0 | 0.00 |
| Urea | 431 | 0 | 0.00 |
| Vasoactive drug use | 431 | 0 | 0.00 |
| White blood cell count | 431 | 0 | 0.00 |

Supplementary Table 2. Final hyperparameter settings, classification thresholds, and class-imbalance handling for the machine learning algorithms

| **Algorithm** | **Final setting** | **Locked threshold** | **Imbalance handling** |
| --- | --- | --- | --- |
| LR | No tuned hyperparameter | 0.3502 | None |
| NB | Laplace=0; kernel=TRUE; adjustment=1 | 0.6776 | None |
| SVM | sigma=0.0625; C=0.25 | 0.3405 | None |
| SHLNN | size=3; decay=0.1 | 0.3143 | None |
| RF | mtry=3; ntree=500 | 0.4425 | None |
| LightGBM | trees=100; depth=2; shrinkage=0.05; min node=10 | 0.4219 | None |
| KNN | kmax=9; Euclidean distance; rectangular kernel | 0.3882 | None |
| DT | cp=0.05 | 0.4926 | None |

Supplementary Table 3. Comparison of the discriminative performance of the random forest model, SOFA score, and APACHE II score for predicting in-hospital mortality in the validation set.

| **Model or score** | **AUC** | **95% CI** | **DeLong P value vs. RF** |
| --- | --- | --- | --- |
| Random forest | 0.804 | 0.730–0.879 | Reference |
| SOFA | 0.613 | 0.514–0.711 | <0.001 |
| APACHE II | 0.690 | 0.598–0.782 | 0.017 |

Abbreviations: RF, random forest; SOFA, Sequential Organ Failure Assessment; APACHE II, Acute Physiology and Chronic Health Evaluation II; AUC, area under the receiver operating characteristic curve; CI, confidence interval. P values were calculated using paired DeLong tests comparing the AUC of each clinical score with that of the RF model.

Supplementary Figure 1. Comparison of receiver operating characteristic curves for the random forest model, SOFA score, and APACHE II score in the validation set。
